# Fruit and vegetable intake is associated with frequency of breakfast, lunch and evening meal: cross-sectional study of 11-, 13-, and 15-year-olds

**DOI:** 10.1186/1479-5868-9-9

**Published:** 2012-02-06

**Authors:** Trine Pagh Pedersen, Charlotte Meilstrup, Bjørn E Holstein, Mette Rasmussen

**Affiliations:** 1 National Institute of Public Health, University of Southern Denmark, Øster Farimagsgade 5, 1353 Copenhagen K, Denmark

**Keywords:** Fruit, Vegetables, Breakfast, Lunch, Evening meal, Adolescents, Sex differences

## Abstract

**Background:**

Frequency of eating breakfast, lunch and evening meal as a determinant of fruit and vegetable intake among young people is little studied. We investigated whether irregular meal consumption was associated with fruit and vegetable intake among adolescents. We used separate analyses, and special emphasis was on the potentially modifying effect of sex and age.

**Methods:**

Data were from the Danish contribution to the international collaborative Health Behavior in School-Aged Children Study (HBSC) in 2002. We used a questionnaire-based, cross-sectional design to study schoolchildren aged 11, 13 and 15 years (n = 3913) selected from a random sample of schools in Denmark. Fruit intake and vegetable intake were measured by a food frequency questionnaire and analyses were conducted using multivariate logistic regression.

**Results:**

Overall, statistically significant associations were found between irregular breakfast, lunch and evening meal consumption and low frequency of fruit intake and vegetable intake (breakfast: fruit OR = 1.42, vegetables OR = 1.48; lunch: fruit OR = 1.68, vegetables OR = 1.83; evening meal: vegetables OR = 1.70). No association was found for irregular evening meal consumption and low frequency of fruit intake. Analyses stratified by sex showed that the associations between irregular breakfast consumption and both fruit and vegetable intake remained statistically significant only among girls. When analyses were stratified by both sex and age, different patterns appeared. Overall, skipping meals seemed to be a less serious risk factor for low frequency of fruit and vegetable intake among younger participants compared with those who were older. This was especially evident for skipping breakfast. The same tendency was also seen for skipping lunch and evening meal, although the age pattern varied between boys and girls and between fruit and vegetable intake.

**Conclusion:**

Our results showed that irregular breakfast, lunch and evening meal consumption among adolescents was associated with a low frequency of fruit and vegetable intake and that sex and age may play a modifying role. The different associations observed in different age and sex groups indicate the importance of analysing fruit and vegetable intake and meal types separately. The results highlight the importance of promoting regular meal consumption when trying to increase the intake of fruit and vegetables among adolescents.

## Background

A sufficient intake of fruit and vegetables is important from a public health perspective. Diets high in fruit and vegetables reduce the risk of cardiovascular disease and some forms of cancer [1-5]. Eating sufficient fruit and vegetables in childhood and adolescence is particularly important. Firstly, during this period the body grows rapidly, requiring many nutrients, which fruit and vegetables can provide [6]. Secondly, food habits established in childhood and adolescence tend to track into adulthood [7,8]. Lastly, during childhood and adolescence there is considerable potential to promote fruit and vegetable intake as food habits are often less consolidated than in adulthood; accordingly, they may be easier to change [9].

The determinants of fruit and vegetable intake among adolescents are numerous [10]. One little-studied factor is the influence of meal frequencies. Sjöberg et al. (2003) found a significantly higher intake of both fruit and vegetables among 15-16-year-old Swedish girls with regular meals. The analysis showed that girls who skipped both breakfast and lunch at least once a week made less healthy food choices than those who ate breakfast and lunch every day. Those who skipped meals consumed less fruit and vegetables and larger amounts of white bread, soft drinks and sweets. The analysis was not conducted for boys [11]. Similarly, a study by Siega-Riz et al. (1998) of American 11-18-year-olds showed that the number of servings of fruit and vegetables (combined measure) was higher among adolescents with frequent meal consumption compared with adolescents with less frequent meal consumption [12]. Melnik et al. (1998) showed that American schoolchildren in 2^nd ^and 5^th ^grade who skipped at least one main meal daily consumed fewer servings of fruit and vegetables (combined measure) than those who did not skip meals. The association did not vary by age. Analyses were controlled by sex [13]. In a Norwegian study among 13-15-year-olds, Lien et al. (2002) constructed a meal score combining weekly frequency of breakfast, lunch and evening meal consumption. They found that a higher meal score was associated with higher intake of fruit and vegetables (combined measure) [14]. Nevertheless, analyses by Neumark-Sztainer et al. (2003) among 3957 American adolescents (mean age 14.9 years) did not reveal any correlation between combined meal frequency and fruit and vegetable intake [15]. Similarly, in a study by Cullen et al. (2004) among 8-10-year-old African-American girls, no association between meal frequency and intake of fruit and vegetables (combined measure) was observed [16].

As demonstrated, the literature on the association between meal frequencies and fruit and vegetable intake among adolescents is limited and inconclusive. Further, there is almost no evidence on the potentially modifying effect of sex and age. Our aim was to investigate whether frequency of breakfast, lunch and evening meal consumption was associated with fruit intake and vegetable intake among adolescents. Further, we aimed to investigate whether potential associations differed between boys and girls and between different age groups.

In the present study fruit intake and vegetable intake and frequency of breakfast, lunch and evening meal consumption were analysed separately. We chose this approach because associations may vary by fruit and vegetables and by meal type.

## Methods

### Study design and study population

We used data from the Danish contribution to the international collaborative cross-sectional study 'Health Behaviour in School-Aged Children (HBSC)' [17]. The purpose of the HBSC study is to increase the understanding of young people's health, wellbeing and health behaviours in their social context. Data collections are conducted every fourth year among schoolchildren aged 11, 13 and 15 years (in Denmark, equivalent to 5^th^, 7^th ^and 9^th ^grade, respectively) from a random sample of schools, i.e. cluster sampling. The schoolchildren complete the self-administered and internationally standardised anonymous HBSC questionnaire at school [18]. The HBSC member countries are involved in a continuous process of developing and validating their research instruments, and validation studies on a wide range of topics have been published [19-26].

No formal agency for ethical approval of school-based surveys exists in Denmark. The HBSC study requests approval for the study through separate letters to the school board (parents' representatives), the school management and the schoolchildren's council in each of the participating schools. The schoolchildren receive written and oral information about the study and are informed that participation is voluntary and anonymous.

In 2002 the Danish sample included 79 schools, of which 68 agreed to participate. The participating schools comprised 5400 schoolchildren in 297 classes. Of these, 4981 schoolchildren were present on the day of data collection, and 4824 schoolchildren submitted a satisfactorily answered questionnaire. The response rate was 89.3%. In the present analyses, adolescents with another ethnic background than Danish were excluded because their diet and meal habits often differ from those with a native Danish background. A previous Danish study found ethnic differences in diets [27]. A newly published Danish report on HBSC data found that adolescents with another ethnic background than Danish ate fruit and vegetables more frequently and skipped breakfast more often than adolescents with a native Danish background did [28]. Further, the group of adolescents in the present study with another ethnic background than Danish was heterogenic, representing 50 different languages, which would have made interpreting the results difficult. A respondent was defined as having another ethnic background than Danish if neither the respondent nor the parents were born in Denmark or if a foreign language was spoken at the respondent's home. After exclusion of respondents with another ethnic background than Danish, the study sample comprised 3913 schoolchildren (1889 boys; 2024 girls). The mean ages of the participants in Grade 5, 7 and 9 were 11.7, 13.7 and 15.7 years, respectively.

### Measurements

#### Dependent variables

We measured the intake of fruit and vegetables separately by a food frequency questionnaire (FFQ) on several food/drink items. The specific wording was: "How many times a week do you usually eat/drink..." followed by a list of different food and drink items. One of the items was "Fruit" and another "Vegetables". Response categories were: "Never", "Less than once a week", "Once a week", "2-4 days a week", "5-6 days a week", "Once a day every day" and "Several times every day". We dichotomised each variable where low intake of fruit and vegetables, respectively, was defined by eating fruit/vegetables 5-6 days a week or less frequently. In Denmark adolescents are recommended to eat 600 g fruit and vegetables daily. The data available for analyses did not provide information on the amount of the adolescents' daily intake. From a nutritional viewpoint, eating fruit and vegetables daily is preferable to eating fruit and vegetables less frequently. Further, we conducted sensitivity analyses by repeating the analyses with different cut points of both the dependent and independent variables. The direction of the associations was the same when changing cut points. The dichotomisation was based upon both a nutritional perspective and statistical power considerations.

#### Independent variables

The independent variables were regularity of breakfast, lunch and evening meal, respectively. The schoolchildren responded to the following three questions: "On WEEKDAYS: How often do you usually have breakfast (more than a glass of milk or juice)/lunch (a proper meal in the middle of the day)/evening meal (a proper meal in the evening)". Response categories were: "I never have breakfast on weekdays", "1 day", "2 days", "3 days", "4 days", "5 days". We dichotomised each independent variable into irregular and regular meal consumption. Irregular breakfast and lunch consumption was defined as consuming the specific meal on 3 weekdays or less frequently. No clear guidelines exist regarding regular meal consumption, but according to the current Nordic Nutrition Recommendations (2004), a regular meal consumption during the day is recommended to avoid frequently eating snacks [29]. From this perspective, a regular meal intake is defined as eating the specific meal every day. However, in the present study, we chose the approach that a meal can occasionally be skipped and meal consumption can still be regular. For the evening meal, the cut point chosen was eating the meal on 4 weekdays or less frequently. This different cut point was applied due to the distribution of the variable.

#### Covariates

We included a number of covariates known to be associated with skipping meals and fruit and vegetable intake among adolescents. These were sex [10,30], age [10,31], weight status [32,33], intended weight loss [33,34], body perception [11,33], family type [10,31,35], and socioeconomic position [10,11,31,36].

Grade (Grade 5, 7 and 9) was used as a proxy for age as the age variation within grades was small. The respondents were asked about their weight without clothes and height without shoes. Weight status (dichotomised into overweight/notoverweight) was defined by BMI cut points set by the International Obesity Task Force [37]. Self-reporting weight and height has limitations as it often leads to an underestimation of BMI [38]. Self-reported data was the only source in the HBSC study. Intended weight loss was measured by the item "Are you on a diet, or do you do other things to lose weight?" ("No, my weight is fine", "No, but I ought to lose weight", "No, because I have to gain weight" and "Yes, I am trying to lose weight"). We dichotomised the variable into respondents who were trying to lose weight against the remaining categories. Body perception was measured by asking the respondents "Do you think your body is...", "Much too thin", "A bit too thin", "Appropriate", "A bit too fat" and "Much too fat". The variable was dichotomized into those who saw themselves as "A bit too fat" and "Much too fat" versus the remaining categories. The variable family type was constructed from the respondents' reports of who they lived with. Respondents were categorised into four categories "Traditional family" (living with two biological parents), "Single parent", "Reconstructed family" (living with mother and stepfather or with father and stepmother) and "Other family types" (e.g. foster homes). The variable family social class was based on the respondent's information about parents' occupation and was trichotomized into: high family social class (Social Class I and II), medium family social class (Social Class III and IV) and low family social class (Social Class V and economically inactive). The categorisations into social classes followed the standards of the Danish National Institute of Social Research [39]. This standard is almost identical to the UK Registrar General's classification.

Table 1 presents the schoolchildren's distribution on the included variables.

**Table 1:** The schoolchildren described by the applied variables, per cent (n)

	Boys						Girls						Total
	*11 years*		*13 years*		*15 years*		*11 years*		*13 years*		*15 years*		
	%	*n*	%	*n*	%	*n*	%	*n*	%	*n*	%	*n*	*n = 3913*
**Dependent variables**													

**Low frequency of fruit intake**^**1**^													
*Yes*	66.2	*(432)*	74.4	*(495)*	81.8	*(467)*	57.3	*(422)*	62.6	*(418)*	63.6	*(394)*	*2628*
*No*	33.1	*(216)*	24.5	*(163)*	16.5	*(94)*	41.3	*(304)*	36.7	*(245)*	35.5	*(220)*	*1242*
*Missing*	0.8	*(5)*	1.1	*(7)*	1.8	*(10)*	1.4	*(10)*	0.8	*(5)*	1.0	*(6)*	*43*
**Low frequency of vegetable intake**^**1**^													
*Yes*	67.5	*(441)*	74.3	*(494)*	78.3	*(447)*	65.2	*(480)*	67.7	*(452)*	71.6	*(444)*	*2758*
*No*	31.6	*(206)*	23.2	*(154)*	19.6	*(112)*	33.0	*(243)*	31.4	*(210)*	27.1	*(168)*	*1093*
*Missing*	0.9	*(6)*	2.6	*(17)*	2.1	*(12)*	1.8	*(13)*	0.9	*(6)*	1.3	*(8)*	*62*

**Independent variables**													

**Breakfast consumption**													
*Irregular^2^*	12.6	*(82)*	15.0	*(100)*	18.7	*(107)*	12.6	*(93)*	23.7	*(158)*	26.8	*(166)*	*706*
*Regular*	86.2	*(563)*	84.5	*(562)*	79.5	*(454)*	86.7	*(638)*	75.5	*(504)*	71.9	*(446)*	*3167*
*Missing*	1.2	*(8)*	0.5	*(3)*	1.8	*(10)*	0.7	*(5)*	0.9	*(6)*	1.3	*(8)*	*40*
**Lunch consumption**													
*Irregular^2^*	24.7	*(161)*	34.1	*(227)*	31.9	*(182)*	17.4	*(128)*	36.1	*(241)*	31.9	*(198)*	*1137*
*Regular*	74.1	*(484)*	65.4	*(435)*	66.4	*(379)*	81.7	*(601)*	63.2	*(422)*	67.3	*(417)*	*2738*
*Missing*	1.2	*(8)*	0.5	*(3)*	1.8	*(10)*	1.0	*(7)*	0.8	*(5)*	0.8	*(5)*	*38*
**Evening meal consumption**													
*Irregular^3^*	6.9	*(45)*	7.7	*(51)*	7.5	*(43)*	5.8	*(43)*	11.5	*(77)*	18.1	*(112)*	*371*
*Regular*	91.9	*(600)*	91.9	*(611)*	91.4	*(522)*	93.6	*(689)*	88.2	*(589)*	81.1	*(503)*	*3514*
*Missing*	1.2	*(8)*	0.5	*(3)*	1.1	*(6)*	0.5	*(4)*	0.3	*(2)*	0.8	*(5)*	*28*

**Covariates**													

**Overweight status**													
*Overweight*	10.6	*(69)*	8.4	*(56)*	13.5	*(77)*	9.7	*(71)*	8.1	*(54)*	9.8	*(61)*	*388*
*Not overweight*	76.0	*(496)*	79.9	*(531)*	78.1	*(446)*	74.2	*(546)*	83.1	*(555)*	83.7	*(519)*	*3093*
*Missing*	13.5	*(88)*	11.7	*(78)*	8.4	*(48)*	16.2	*(119)*	8.8	*(59)*	6.5	*(40)*	*432*
**Intended weight loss**													
*Yes*	16.9	*(110)*	13.2	*(88)*	10.9	*(62)*	22.8	*(168)*	30.1	*(201)*	35.3	*(219)*	*848*
*No*	82.2	*(537)*	85.7	*(570)*	87.7	*(501)*	75.4	*(555)*	68.3	*(456)*	62.9	*(390)*	*3009*
*Missing*	0.9	*(6)*	1.1	*(7)*	1.4	*(8)*	1.8	*(13)*	1.7	*(11)*	1.8	*(11)*	*56*
**See oneself as fat**													
*Yes*	25.6	*(167)*	25.7	*(171)*	22.9	*(131)*	33.0	*(243)*	44.0	*(294)*	48.2	*(299)*	*1305*
*No*	73.7	*(481)*	73.5	*(489)*	74.8	*(427)*	66.4	*(489)*	55.2	*(369)*	50.5	*(313)*	*2568*
*Missing*	0.8	*(5)*	0.8	*(5)*	2.3	*(13)*	0.5	*(4)*	0.8	*(5)*	1.3	*(8)*	*40*
**Family type**													
*Traditional*	60.5	*(395)*	62.4	*(415)*	66.0	*(377)*	60.3	*(444)*	58.7	*(392)*	58.7	*(364)*	*2387*
*Single parent*	14.6	*(95)*	15.2	*(101)*	13.7	*(78)*	13.5	*(99)*	15.3	*(102)*	13.6	*(84)*	*559*
*Reconstructed*	11.2	*(73)*	11.9	*(79)*	9.8	*(56)*	12.9	*(95)*	12.0	*(80)*	12.7	*(79)*	*462*
*Others*	1.8	*(12)*	1.2	*(8)*	1.1	*(6)*	1.2	*(9)*	2.0	*(13)*	2.3	*(14)*	*62*
*Missing*	11.9	*(78)*	9.3	*(62)*	9.5	*(54)*	12.1	*(89)*	12.1	*(81)*	12.7	*(79)*	*443*
**Family social class**													
*High*	21.9	*(143)*	19.6	*(130)*	30.1	*(172)*	21.1	*(155)*	20.1	*(134)*	25.5	*(158)*	*892*
*Medium*	47.0	*(307)*	52.3	*(348)*	49.2	*(281)*	51.2	*(377)*	55.7	*(372)*	53.7	*(333)*	*2018*
*Low*	17.9	*(117)*	16.1	*(107)*	15.1	*(86)*	16.9	*(124)*	15.1	*(101)*	15.3	*(95)*	*630*
*Missing*	13.2	*(86)*	12.0	*(80)*	5.6	*(32)*	10.9	*(80)*	9.1	*(61)*	5.5	*(34)*	*373*

^1^Eat fruit/vegetables 5-6 days a week or less ^2 ^Eat breakfast/lunch three weekdays or less

^3^Eat evening meal four weekdays or less

#### Statistical analyses

In the descriptive analysis, we tested sex and age differences by chi^2 ^tests and used Cochran's test to test for trend. We used multivariate logistic regression analyses to examine the association between meal consumption and frequency of fruit and vegetable intake. Initial analyses included the full study population. In the next step the analyses were stratified by sex to assess whether sex was an effect modifier (moderator). To evaluate the modifying effect of age, the final analyses were also stratified by age. Additionally, the modifying effect of sex and age was tested by including an interaction term in the statistical models.

The logistic regression analyses were modelled against low frequency of fruit intake and low frequency of vegetable intake, respectively. Covariates were included in the analyses, and to avoid losing statistical power, missing data remained in the analyses. Before including the respondents with missing data, we observed that respondents without and with missing data differed substantially against the dependent variable. Generally, the group of missing data was placed in the reference group to counteract an exaggeration of statistical associations. For covariates with a significant proportion of missing values (family social class, weight status and family type), missing data were kept as a separate category, see Table 1. We chose this approach for the three covariates because we found it difficult to place the large amount of missing data in any of the covariate categories. We conducted the logistic regressions in PROC GLIMMIX to adjust for the potential design effect introduced by the cluster sampling design (the unit of sampling was schools and school classes). Separate inclusion of random effects of school and school class revealed nearly identical estimates. Analyses including the random effect of school were reported. For some strata the PROC GLIMMIX procedure did not converge. In these cases we conducted PROC LOGISTIC with schools included in the analyses as a covariate. School was defined as a class variable and thereby a dummy variable was included in the analyses for each school included. We performed sensitivity analyses by repeating the analyses with different cut points of both the dependent and independent variables. The direction of the associations was the same when changing cut points. Generally, choice of extreme cut points resulted in stronger associations, and choice of cut points closer to the median resulted in weaker associations, in some cases even statistically insignificant associations. As mentioned in the measurement section, the cut points were chosen from a nutritional perspective and with regard to the statistical limitations. We excluded adolescents with another ethnic background than Danish in the analyses because of the existing literature regarding ethnic differences in skipping breakfast and fruit and vegetable intake. We repeated the analyses including the group of adolescents with another ethnic background than Danish and the estimates did not change direction.

## Results

### Descriptive analysis

We found that the proportion of schoolchildren with low frequency of fruit intake was statistically significantly larger among boys than among girls (boys: 73.8%; girls: 61.0%, *p *< 0.0001). The same pattern was seen for vegetable intake (boys: 73.2%; girls: 68.0%, *p *< 0.0001). Significantly more girls than boys skipped breakfast (girls: 20.6%; boys: 15.3%, *p *< 0.0001) and the evening meal (girls: 11.5%; boys: 7.4%, *p *< 0.0001).

Among boys, the proportion with a low frequency of fruit intake and vegetable intake, and with irregular breakfast consumption increased with increasing age (trend test respectively: *p *< 0.0001; *p *< 0.0001; *p *= 0.0024). Only for fruit intake was the age gradient stepwise statistically significant. Among girls, we found an age gradient similar to that of boys for low frequency of fruit intake, vegetable intake, along with irregular breakfast and evening meal consumption (trend test respectively: *p *= 0.0210; *p *= 0.0164; *p *< 0.0001; *p *< 0.0001). However, the age gradient was stepwise statistically significant only for irregular evening meal consumption.

A significantly larger proportion of girls than boys reported that they were trying to lose weight (girls: 29.1%; boys: 13.8%, *p *< 0.0001). For body perception, a significantly larger proportion of girls than boys perceived their body as fat (girls: 41.3%; boys: 24.8%, *p *< 0.0001).

### Multivariate analysis

#### Breakfast consumption and fruit intake

Among the full study population, we found that irregular breakfast consumption was associated with low frequency of fruit intake (Table 2). When we stratified the analyses by sex, the association was not found among boys. There was no statistically significant interaction with sex. Among boys, the association between irregular breakfast consumption and frequency of fruit intake showed a statistically significant interaction with age (p = 0.0011). Among 11-year-old boys, irregular breakfast consumption was significantly and inversely associated with low frequency of fruit intake (OR = 0.52 (0.30-0.90)); irregular breakfast consumption was strongly associated with low fruit intake among 13-year-olds (OR = 4.99 (1.90-13.07)). We found the same pattern among the 15-year-old boys, albeit statistically insignificant (OR = 1.69 (0.69-4.13)).

Among girls, the association became statistically insignificant for irregular breakfast consumption among 11-year-olds (OR = 1.55 (0.81-2.96)). However, no statistically significant interactions with age were found.

**Table 2:** Adjusted OR (CI 95%) for low fruit intake by frequency of breakfast, lunch and evening meal, separately for boys and girls in three age groups. Adjusted for covariates

	All schoolchildren	Boys	Girls	Sex interaction	Boys			Age interaction	Girls			Age interaction
				***P*-value**	**11 years**	**13 years**	**15 years**	***P*-value**	**11 years**	**13 years**	**15 years**	***P*-value**

**Breakfast**												
Irregular vs. regular	**1.42** **(1.12-1.79)**	1.12 (0.77-1.63)	**1.63** **(1.21-2.21)**	0.1609	**0.52** **(0.30-0.90)**	**4.99** **(1.90-13.07)**^**A**^	1.69 (0.69-4.13)^**A**^	**0.0011**	1.55 (0.81-2.96)	**1.80** **(1.08-3.01)**	**1.64** **(1.02-2.61)**	0.9574
**Lunch**												
Irregular vs. regular	**1.68** **(1.39-2.04)**	**1.95** **(1.44-2.63)**	**1.49** **(1.16-1.92)**	0.2099	1.26 (0.78-2.04)	**2.46** **(1.36-4.45)**^**A**^	**4.11** **(1.89-8.93)**^**A**^	**0.0423**	**1.70** **(1.00-2.88)**	1.38 (0.92-2.09)	**1.54** **(1.01-2.37)**	0.8220
**Evening meal**												
Irregular vs. regular	1.24 (0.93-1.66)	1.23 (0.74-2.07)	1.30 (0.92-1.83)	0.9120	0.98 (0.44-2.14)	1.11 (0.44-2.76)^**A**^	7.62 (0.89-65.05)^**A**^	0.2028	1.01 (0.47-2.17)	1.66 (0.87-3.17)	1.21 (0.72-2.02)	0.6605

^A^The analyses were performed using PROC LOGISTIC because PROC GLIMMIX did not converge

The analyses including the full study population were adjusted for the covariates: sex, age, intended weight loss, body perception, overweight, family type and family social class

The sex stratified analyses were adjusted for the same covariates except sex

The sex and age stratified analyses were adjusted for the same covariates except sex and age

#### Lunch consumption and fruit intake

In the logistic regression analyses of the full study population, we found that irregular lunch consumption was associated with low frequency of fruit intake (Table 2). The association between irregular lunch consumption and frequency of fruit intake among boys was modified by age (*p *= 0.0423). Here, increasing ORs were observed with increasing age (11-year-olds: OR = 1.26 (0.78-2.04); 13-year-olds: OR = 2.46 (1.36-4.45); 15-year-olds: OR = 4.11(1.89-8.93). Among girls, the association became statistically insignificant for irregular lunch consumption among 13-year-olds (OR = 1.38(0.92-2.09)). However, we found no statistically significant interactions with age among girls.

#### Evening meal consumption and fruit intake

In the logistic regression analyses, we found no association between irregular evening meal consumption and frequency of fruit intake (Table 2).

#### Breakfast consumption and vegetable intake

In our analyses of the full study population, a statistically significant association was observed between irregular breakfast consumption and low frequency of vegetable intake (Table 3). There was no statistically modifying effect of sex, although a tendency was observed in the estimates (boys: OR = 1.19 (0.82-1.73); girls: OR = 1.78 (1.28-2.47). Additionally, we found no statistically significant interactions with age. However, among 11-year-old boys, skipping breakfast was not associated with low vegetable intake, OR = 0.95 (0.53-1.70), while irregular breakfast consumption showed a high OR among 15-year-olds, OR = 2.67 (1.16-6.09). Among girls, we found a tendency of increasing OR with increasing age for low frequency of vegetable intake. Only for 15-year-old girls did we find a statistically significant association, OR = 2.87 (1.61-5.10) and the statistical interaction was significant at a 10% level (*p *= 0.0874).

**Table 3:** Adjusted OR (CI 95%) for low vegetable intake by frequency of breakfast, lunch and evening meal, separately for boys and girls in three age groups. Adjusted for covariates

	All schoolchildren	Boys	Girls	Sex interaction	Boys			Age interaction	Girls			Age interaction
				**P-value**	**11 years**	**13 years**	**15 years**	**P-value**	**11 years**	**13 years**	**15 years**	**P-value**

**Breakfast**												
Irregular vs. regular	**1.48** **(1.16-1.89)**	1.19 (0.82-1.73)	**1.78** **(1.28-2.47)**	0.1239	0.95 (0.53-1.70)	1.00 (0.45-3.70)^**A**^	**2.67** **(1.16-6.09)**^**A**^	0.2341	1.09 (0.56-2.14)	1.60 (0.94-2.74)	**2.87** **(1.61-5.10)**	0.0874
**Lunch**												
Irregular vs. regular	**1.83** **(1.50-2.24)**	**1.73** **(1.29-2.31)**	**1.90** **(1.44-2.51)**	0.7684	**1.81** **(1.09-3.02)**	1.48 (0.84-2.60)^**A**^	**2.03** **(1.10-3.77)**^**A**^	0.9833	**2.13** **(1.18-3.83)**	**1.57** **(1.01-2.44)**	**2.02** **(1.24-3.28)**	0.7987
**Evening meal**												
Irregular vs. regular	**1.70** **(1.23-2.33)**	**2.17** **(1.19-3.94)**	**1.51** **(1.03-2.21)**	0.3716	1.26 (0.54-2.90)	**4.35** **(1.16-16.28)**^**A**^	4.00 (0.76-21.01)^**A**^	0.2314	1.36 (0.59-3.11)	1.54 (0.77-3.09)	1.45 (0.81-2.59)	0.9443

^A^The analyses were performed using PROC LOGISTIC because PROC GLIMMIX did not converge

The analyses including the full study population were adjusted for the covariates: sex, age, intended weight loss, body perception, overweight, family type and family social class

The sex stratified analyses were adjusted for the same covariates except sex

The sex and age stratified analyses were adjusted for the same covariates except sex and age

#### Lunch consumption and vegetable intake

In our analyses of the full study population, we found a statistically significant association between irregular lunch consumption and low frequency of vegetable intake (Table 3). There was no statistically modifying effect of sex or age. For girls, we found statistically significant associations in all three age groups (11-year-olds OR = 2.13 (1.18-3.83); 13-year-olds OR = 1.57 (1.01-2.44); 15-year-olds OR = 2.02 (1.24-3.28).

#### Evening meal consumption and vegetable intake

Our logistic regression analyses of the full study population showed that irregular evening meal consumption was associated with a low frequency of vegetable intake (Table 3). Stratification on sex and age revealed no statistically significant interactions, but we observed a high OR among 13-year-old boys, OR = 4.35 (1.16-16.28) and the same pattern was seen among 15-year-old boys, although statistically insignificant, OR = 4.00 (0.76-21.01). Among the 11-year-old boys, the OR was 1.26 (0.54-2.90). We found no association between irregular evening meal consumption and frequency of vegetable intake in any of the age-stratified analyses among girls.

## Discussion

We found that there was an overall association between irregular consumption of breakfast, lunch and evening meal and low frequency of fruit and vegetable intake. Only for irregular evening meal consumption and low frequency of fruit intake was no association found. Analyses stratified by sex showed that the associations between irregular breakfast consumption and both fruit and vegetable intake remained statistically significant only among girls. When the data were stratified on sex and age, different patterns appeared. In general terms, skipping meals seemed to be a less serious risk factor for low frequency of fruit and vegetable intake among younger schoolchildren compared with those who were older. This was especially evident for skipping breakfast. The same tendency was also seen for skipping lunch and evening meal, although the age pattern varied between boys and girls and between fruit and vegetable intake.

Earlier studies have documented an association between meal frequencies and fruit and vegetable intake among adolescents [11-14]. These studies support our results, despite the different measurement methods regarding meal consumption and fruit and vegetable intake. We have not identified any studies which examine the modifying effect of sex. An earlier American study by Melnik et al. (1998) found that American children who skipped meals had fewer servings of fruit and vegetables and they found the same association in all age groups [13]. In the present study, we found several variations in the association between skipping meals and fruit and vegetable intake between different age groups. There are, however, different reasons why the results of the present study are not fully comparable with the findings of Melnik et al. (1998). In the American study the outcome measure was a combined scale of fruit and vegetable intake. Additionally, the age groups included were younger compared with those in the present study. Finally, in the analyses of American children, stratification by sex was not conducted. It has not been possible to find studies examining the modifying effect of both sex and age on the association between irregular meal consumption and intake of fruit and vegetables.

The observed modifying effect of age in the analyses showed that skipping meals seemed to be a less serious risk factor for low frequency of fruit and vegetable intake among younger schoolchildren compared with older schoolchildren. A possible explanation could be that younger children, despite skipping meals, are still offered fruit and vegetables on other occasions and that their parents have some form of control over what they eat. When children become older, they become increasingly independent of their parents, and the family influences on eating behaviour diminish [40].

The lack of statistically significant associations between irregular evening meal consumption and frequency of vegetable intake in the analyses stratified by age can be explained by insufficient statistical power. In a Danish context, an association between irregular evening meal consumption and low frequency of vegetable intake was to be expected as vegetables, in particular, are included in the evening meal [41]. This hypothesis was confirmed by statistically significant associations in the analyses stratified by sex. Further, although insignificant, support was also provided by relatively strong odds ratios in our age stratified analyses.

Yngve et al. (2005) found that Nordic children have a higher consumption of raw vegetables compared with children in other European countries [42]. In this study we found that the intake of vegetables had a strong association with the consumption of lunch among both boys and girls in all three age groups. This indicates that the children ate vegetables at lunch. This is consistent with previous descriptions of Danish adolescents' meal habits [41]. The intake of vegetables did not seem to be associated with consumption of breakfast. This pattern is to be expected as vegetables do not contribute to a typical Danish breakfast [41]. Nevertheless, among the 15-year-old girls, we found a strong association between irregular breakfast consumption and low frequency of vegetable intake. This association suggests that skipping meals in general is an indicator of having unhealthy eating habits. Other studies have shown an association between poor nutrition and skipping breakfast [32,43].

### Strengths and limitations

The main strengths of the present study are the inclusion of a large national representative sample of young people and the application of a well-documented and widely used instrument. Another strength is the use of separate measures of fruit and vegetable intake and separate measures for the three meal types.

We modelled breakfast, lunch and evening meal consumption as independent variables and fruit and vegetable intake as dependent variables. However, due to the cross-sectional design of the study, causality cannot be assumed. Potentially, intake of fruit and vegetables could influence the consumption of meals.

The response rates were relatively high at both the school level and the individual level. The non-participating schools' reasons for not participating did not indicate that non-participating schools differed by central variables or by covariates included in analyses. Nonetheless, non-participating schools and schoolchildren may have resulted in selection bias. A detailed analysis of non-participating children has not been possible due to the complete anonymity of the study.

In the present study, adolescents with another ethnic background than Danish were excluded because their diet and meal habits differ substantially from the adolescents with a native Danish background [27,28]. Accordingly, it is not possible to generalise the findings to the ethnic minorities in the country.

The literature shows varying results regarding the validity of self-reported dietary assessment methods among adolescents [26,44-48]. Some studies have recognized FFQ as a valid instrument for ranking adolescents according to their usual intake [26,44]. Thus, although not being suitable for estimating prevalence, data generated by FFQs may be applicable for aetiological studies. When using FFQs, potential information bias due to recall bias must be considered. There is also a risk of social desirability bias where respondents report a socially acceptable intake instead of the true intake [49]. Studies find that an increased level of information about healthy food may contribute to an over-reporting of intake of fruit and vegetables [49-51]. In the HBSC FFQ measure, there is no definition of fruit and vegetables. Therefore we do not know whether the children included fruit and vegetables in beverages. This could introduce an information bias to the study.

It is a limitation of the study that we used a single question for measuring meal frequencies and the definition of a meal is sparse in the question formulation. The question on breakfast frequency was validated in an HBSC study in 2004-2005. The validation showed a moderate accordance with food-habit diaries (kappa statistics 0.47) [52]. Another limitation is that our measure of frequency of meals included only weekdays. We chose this because weekend routines and practices regarding meals and diet might differ from those of weekdays. Previous research has found that people have a higher caloric intake and eat larger servings during weekends [53-55].

For unmeasured confounding, an underlying factor affecting the observed associations could be the family's general concern about eating- and meal habits. Other studies have found a positive association between family meals and children's intake of fruit, vegetables, and breakfast [10,56,57]. The association between family meals and intake of fruit and vegetables may reflect an overall positive home food environment with higher availability of fruit and vegetables and support for healthy meal habits [57].

## Conclusions

The results showed that irregular consumption of breakfast, lunch and evening meal was associated with low intakes of fruit and vegetables among adolescents. Stratified analyses showed a strong association among 15-year-olds of both sexes and among girls. The different patterns in our analyses indicate that it is important to use separate measures of fruit and vegetable intake and separate measures for the three meal types. We focused on a homogeneous population of native Danish children; future studies are needed with sufficiently large proportions of immigrants to study the role of cultural diversity in the association between meal frequency and consumption of fruit and vegetables. For future studies, it is important to develop more refined and validated measurements of meal habits.

From a public health perspective, the results of the present study indicate the relevance of promoting regular meal consumption as part of an overall strategy for healthy nutritional habits among adolescents, including increased intake of fruit and vegetables. This study also showed that there is a need for tailored programmes aiming at boys and girls of different ages. Adolescents consume their meals in both the family setting and school setting. For the family setting, Videon & Manning (2003) propose interventions to improve adolescent nutrition. They stress the significance of parents emphasizing the importance of healthy breakfast habits and providing guidelines for their children [56]. For the school setting, one way to promote healthy meal habits could be to increase the availability and quality of school meals. Participation in school lunch programmes that include fruit and vegetables has been found to be associated with a higher fruit and vegetable intake [13].

## Competing interests

The authors declare that they have no competing interests.

## Authors' contributions

TPP, CM, BEH and MR contributed to the planning of research questions and analytical strategy. Data analyses were conducted by TPP and CM. TPP drafted the manuscript with critical input from CM, BEH and MR. All authors have read and approved the final manuscript.
